# Beyond the Norm: Unveiling a Dermoid Cyst in an Inguinal Hernia Case

**DOI:** 10.7759/cureus.43736

**Published:** 2023-08-19

**Authors:** Saleh A Ba-shammakh, Motasem Almaletti, Batool A Aqel

**Affiliations:** 1 Department of General Surgery, The Islamic Hospital, Amman, JOR

**Keywords:** benign skin lesions, germ layers, inguinal swelling, dermoid cyst, inguinal hernia, epidermoid cyst

## Abstract

This case report focuses on a rare presentation of *a dermoid cyst *within an inguinal hernia in a 15-year-old male patient. The patient presented with a four-day history of a persistent, non-reducible, physical exertion-related bulge in the left groin area, which improved upon lying supine. Ultrasound investigations revealed a complex cystic structure resembling a dermoid cyst within a left direct inguinal hernia. Post-operative histopathology confirmed the dermoid cyst with no signs of malignancy. This case emphasizes the need for a high degree of suspicion when dealing with inguinal swellings, as rare entities like epidermal cysts may mimic more common conditions like inguinal hernias.

## Introduction

Epidermoid cysts, commonly termed epidermal cysts, are benign skin lesions that can appear in various body regions, notably the forehead, scalp, face, neck, and trunk. Their development stems from the folding in of the keratinized squamous epithelium, either due to a natural epidermal shift into the dermis or from injury and surgeries [1]. While it's relatively standard to detect intra-abdominal organs, including sections of the bowel, within an inguinal hernia, finding an epidermoid cyst within such a hernia is notably rare [2].

Additionally, identifying a dermoid cyst or mature teratoma within the inguinal canal is uncommon. These cystic formations are congenital, containing mature tissues derived from at least two primary germ layers. While primarily observed within the gonads, these cysts also appear in specific central body parts such as the anterior mediastinum, para-coccygeal zones, pineal region, and certain intracranial, neck, and abdominal areas [3].

The inguinal canal might occasionally house other unique entities like teratomas, lipomas, lymphangiomas, leiomyomas, and endometriosis. Due to their rarity, diagnosing epidermal cysts in the inguinal region is pivotal. This report delves into these atypical findings and discusses their clinical relevance.

## Case presentation

A 15-year-old male patient, previously healthy, presented to the emergency department. For the past four days, he has suffered from a continuous bulge in his left groin area. This swelling would intensify when he lifted heavy objects, but it eased when he lay supine. The patient did not observe any alterations in the skin above the bulge. Furthermore, he denied experiencing constipation, nausea, or vomiting symptoms.

Before coming to the emergency department, the patient hadn't sought medical attention for his condition or been prescribed any medications. As the bulge continued to persist and worsen with physical exertion, he needed professional medical advice.

Upon presentation, the patient was fully conscious, alert, and oriented. His vital signs were as follows: T: 36.6 C, HR: 75 BPM, BP: 110/65 mmHg, and O2 saturation: 98%. The physical examination was largely unremarkable, except for a noticeable bulge in the left groin area, consistent with an inguinal hernia.

**Figure 1:**
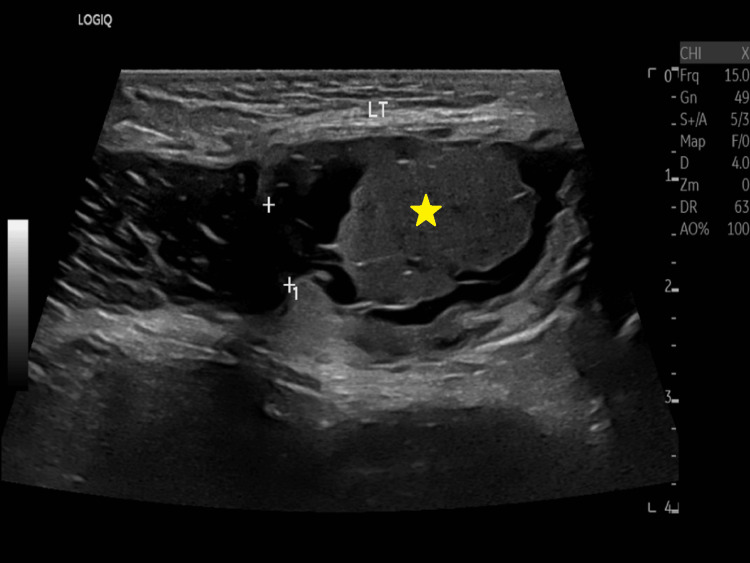
Ultrasound examination of the left groin Demonstrating a structure containing bowel and fluid medial to the inferior epigastric artery and a well-defined complex cystic structure exhibiting mixed echogenicity, measuring approximately 4.2 x 2.2 cm.

An ultrasound examination of the left groin revealed a structure containing bowel and fluid medial to the inferior epigastric artery. This structure was exerting a mass effect on the left spermatic cord, indicating the presence of a left direct inguinal hernia. Additionally, a well-defined complex cystic structure was identified, exhibiting mixed echogenicity similar to subcutaneous fat and a dash and dot appearance, measuring about 4.2 x 2.2 cm (Figure 1).

These characteristics raised suspicion of an accompanying dermoid cyst.

**Figure 2:**
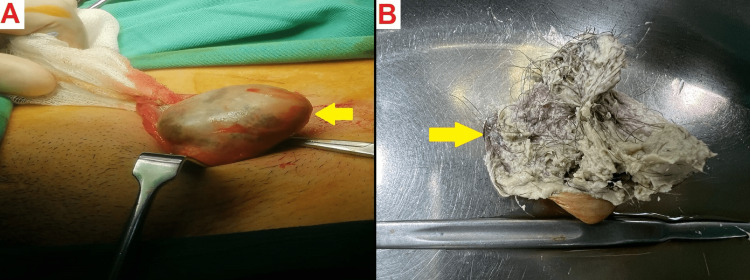
Intraoperative view A: The suspected dermoid cyst. B: A cut section of the cyst shows the presence of greasy material with hair content.

Upon diagnosis, the patient was immediately admitted to the hospital. A successful surgical procedure was performed, which included an incisional repair of his left inguinal hernia. To reinforce the repair, a mesh was used. In the same surgical session, a suspected dermoid cyst identified in the left inguinal region was also removed (Figure 2).

Postoperatively, the patient was mobile, urinated, tolerated his diet, and maintained a clean surgical dressing. His vital signs remained stable within normal limits. An antibiotic treatment regimen with Amoclan was initiated and continued, in addition to Panadol and Diclofenac sodium, for pain management.

**Figure 3:**
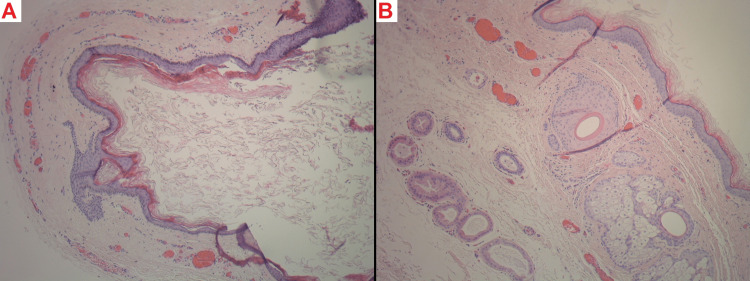
Histopathological examination of the excised tissue A: A cystic space is lined by keratinizing stratified squamous epithelium, and the lumen is filled with loose keratin flakes. H&E stain, 10X magnification. B: The wall of the cyst harbors variable skin appendages, including pilosebaceous units and apocrine ducts, consistent with a dermoid cyst. H&E stain, 10X magnification.

The excised tissue was sent for histopathological examination. The pathological findings confirmed the presence of the suspected dermoid cyst, characterized by a gray-white cyst containing whitish, cheesy material and hair (Figure 3 ).

Notably, there were no signs of malignancy at the levels examined.

Following a satisfactory recovery period, the patient was discharged the next day. He was instructed to continue the prescribed medication regimen, which included Amoclan, Panadol, and Diclofenac sodium, and was scheduled for regular follow-up appointments one week post-discharge. These follow-up appointments aimed to monitor his recovery process closely, check for potential complications, and screen for any recurrence. As of his most recent follow-up, the patient displayed satisfactory healing, with no signs of complications or recurrence.

## Discussion

Epidermoid cysts are recognized for their benign nature. Their presence within an inguinal hernia, however, is an uncommon finding, as demonstrated in our patient's case. These cysts commonly emerge due to the incomplete separation of the ectoderm from the neural tube or when the surface ectoderm fails to properly isolate at embryonic fusion sites. They are mainly classified as either congenital or acquired [4-5]. Diagnosing such cysts necessitates a high degree of suspicion because of their rare appearance in the inguinal region. Due to similarities in clinical presentations, they are often mistakenly diagnosed as hernial sacs containing omentum, irreducible or incarcerated hernias, or lipomas stemming from the spermatic cord [6].

Our patient exhibited a non-reducible, persistent inguinal swelling devoid of a cough impulse. This presentation, coupled with the patient's history and ultrasound findings, hinted at a dermoid cyst. The suspicion was later confirmed via histopathological examination [7]. Crafting a differential diagnosis for inguinal swellings is challenging. The list of possible conditions is vast, spanning femoral hernias, cryptorchidism, lymph node enlargements, and others like lipomas, saphena varix, venous varicosities, and round ligament endometriosis [6].

Both dermoid and epidermoid cysts fall under inclusion cysts lined by the ectoderm. Their distinction lies in their histological makeup: dermoid cysts contain mature tissues from at least two primary germ layers [8]. While dermoid cysts incorporate elements like skin, its appendages, and the sebaceous gland, epidermoid cysts consist of stratified squamous epithelium surrounded by a fibrous tissue wall [8].

In females, dermoid cysts are predominantly ovarian, but they can occasionally be found in the inguinal region in males, as was the case with our patient [9-10]. These lesions, known for their slow growth, often remain undetected until the individual reaches their second or third decade [10]. Imaging techniques, such as ultrasonography, CT, and MRI, play a crucial role in the accurate identification of these cysts. For instance, dermoid cysts usually present as well-defined, unilocular cystic abnormalities on these scans. Elements like fat, soft tissue, and calcifications can be discerned using CT or MRI [11].

For both epidermoid and dermoid cysts, the standard management procedure is surgical excision, as we performed in our case. This treatment method underscores the significance of a correct diagnosis, ensuring that patients do not undergo unnecessary and extensive surgeries for benign conditions.

This case illuminates the value of meticulous clinical evaluations paired with strategic diagnostic modalities to discern common presentations from rarer ones. Unearthing a dermoid cyst in an inguinal hernia should prompt clinicians to exercise heightened vigilance when diagnosing and managing analogous cases [12-13]. Nevertheless, a larger volume of case studies and research is essential to formulating precise diagnostic guidelines and treatment strategies for such unique clinical situations.

## Conclusions

Dermoid cysts in the inguinal region, due to their rarity and resemblance to more common inguinal swellings, can be overlooked. This case report underscores the importance of considering such entities in differential diagnoses. Thorough clinical evaluation, alongside imaging tools, is essential for identifying these rare presentations. The primary treatment is surgical excision, followed by histopathological evaluation to confirm the diagnosis and rule out malignancy. Regular follow-ups ensure successful recovery and monitor potential recurrence.

## References

[1] Handa U, Kumar S, Mohan H (2002). Aspiration cytology of epidermoid cyst of terminal phalanx. Diagn Cytopathol.

[2] Kirkham N (2015). Tumors and cysts of the epidermis. Lever’s Histopathology of the Skin.

[3] Engel RM, Elkins RC, Fletcher BD (1968). Retroperitoneal teratoma. Review of the literature and presentation of an unusual case. Cancer.

[4] EP WL, KL AM (1957). Epithelial cysts in buried human skin. AMA Arch Derm.

[5] Papanayotou PH, Kayavis JG (1977). Epidermoid implantation cyst of the lower lip: report of case. J Oral Surg.

[6] Farrands PA, Taylor I (1984). Unusual groin swellings. Postgrad Med J.

[7] Kn R, Kumar S, Gahlawat S, Agarwal S, Kaushik S (2008). Dermoid cyst of spermatic cord: an uncommon presentation in a child. Annals of Diagnostic Paediatric Pathology.

[8] Smirniotopoulos JG, Chiechi MV (1995). Teratomas, dermoids, and epidermoids of the head and neck. Radiographics.

[9] Genetzakis M, Lagoudianakis EE, Papadima A, Tsekouras DK, Markogiannakis H, Filis K, Manouras A (2006). Inguinal dermoid cyst of the round ligament. A case report and review of the literature. Clin Exp Obstet Gynecol.

[10] Usta IM, Khoury NG, Khalil AM, Nassar AH (2006). Coexistence of a round ligament dermoid cyst and struma ovarii in pregnancy. Eur J Obstet Gynecol Reprod Biol.

[11] Buy JN, Ghossain MA, Moss AA (1989). Cystic teratoma of the ovary: CT detection. Radiology.

[12] Surriah MH, Quttiba AM Dermoid cyst of the spermatic cord: a rare case of benign inguinal lump. Ira Postg Med Jr.

[13] Pfeifer JD, Dehner LP, Humphrey PA (2019). The Washington Manual of Surgical Pathology. https://www.wolterskluwer.com/en/solutions/ovid/washington-manual-of-surgical-pathology-the-4636.

